# Endoscopic yield of chronic dyspepsia in outpatients: A single‐center experience in Cambodia

**DOI:** 10.1002/jgh3.12210

**Published:** 2019-06-24

**Authors:** Borathchakra Oung, Khang Chea, Chakravuth Oung, Jean‐Christophe Saurin, Cynthia W Ko

**Affiliations:** ^1^ Gastroenterology and Endoscopy Unit, Edouard Herriot Hospital Hospices civils de Lyon Lyon France; ^2^ CHAKRA GI Clinic Phnom Penh Cambodia; ^3^ GI and Liver Unit Calmette Hospital Phnom Penh Cambodia; ^4^ Faculty of Medicine University of Health Sciences Phnom Penh Cambodia; ^5^ Cambodian Association of Gastrointestinal Endoscopy Seattle Washington USA; ^6^ Division of Gastroenterology University of Washington Seattle Washington USA

**Keywords:** functional dyspepsia, helicobacter pylori, upper endoscopy, gastric cancer

## Abstract

**Background and Aim:**

The diagnostic evaluation and management of patients with chronic dyspepsia may differ geographically according to patient age, prevalence of *Helicobacter pylori* or parasitic infection, and risk of gastric cancer. The characteristics and appropriate investigation of Cambodian patients with dyspepsia have not previously been studied. The aim of this study was to investigate the characteristics of Cambodian patients with chronic dyspepsia, the yield of upper endoscopy in these patients, and the value of alarm features in identifying patients with organic causes of dyspepsia.

**Methods:**

We conducted a retrospective, single‐center study of 1231 adults with chronic dyspepsia who underwent upper endoscopy. We compared clinical characteristics, *H. pylori* prevalence, and endoscopic and histological findings of patients with functional or organic causes of dyspepsia. This study was approved by the National Ethics Committee for Health Research.

**Results:**

The majority of patients had overlapping symptoms of epigastric pain/burning and postprandial fullness/early satiety (40.6%), followed by epigastric pain/burning alone (29.7%) and postprandial fullness/early satiety alone (29.7%). Organic lesions were diagnosed in 6.9% of patients. The overall prevalence of *H. pylori* infection was 46% and was similar in the three clinical subgroups. The sensitivity and specificity of alarm features for organic causes of dyspepsia were 14 and 96%, respectively. The majority of patients with gastric cancer were 40 years of age or older.

**Conclusions:**

The majority of patients with chronic dyspepsia seen at our outpatient center were diagnosed with functional or *H. pylori*‐associated dyspepsia. The presence of alarm features was not sensitive or specific for differentiating organic and functional dyspepsia.

## Graphical abstract

This paper is the first to describe the clinical and endoscopic correlates of patients with dyspepsia in Cambodia, including predictors for organic causes of dyspepsia such as peptic ulcer disease or gastric cancer. The results are synthesized into a proposed algorithm for the management of dyspepsia in Cambodia.

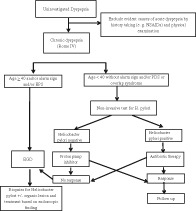

## Introduction

Dyspepsia is a common symptom with an extensive differential diagnosis and a heterogeneous pathophysiology.^1^, ^2^ It occurs in approximately 10–30% of the population each year and has negative impacts on the quality of life and work,^3^ with high direct or indirect costs.^4^, ^5^ Differences in the prevalence of *Helicobacter pylori (H. pylori)*, the availability of diagnostic tests such as noninvasive assays for *H. pylori* or esophagogastroduodenoscopy (EGD), and the risk of gastric cancer influence the diagnostic workup and management of dyspepsia in different countries.^6^, ^7^

The Rome Foundation criteria (Rome IV) subclassify dyspepsia as epigastric pain syndrome (EPS) or postprandial distress syndrome (PDS) to simplify the intricate heterogeneity of the symptom complex and to guide the treatment.^8^ The Kyoto global consensus meeting also distinguishes between *H. pylori*‐associated dyspepsia and functional dyspepsia.^9^ However, symptoms and alarm features cannot distinguish between organic and functional disease.^10^ In Asian countries, infestation by certain parasites may also cause dyspepsia. An Asian consensus statement recommended that, in areas with high prevalence of parasitic infestations, a stool examination may identify parasites such as *Ascariasis*, *Fascioliasis*, *Giardiasis*, and *Opisthorchiasis* that can cause dyspeptic symptoms.^11^ Many studies have shown a high prevalence of parasitic infestation in Cambodia.^12^

The most concerning cause of dyspepsia is upper gastrointestinal malignancy. The risk of upper gastrointestinal malignancy is heterogeneous in Asia, ranging from low (Bangladesh, India, Thailand) and average (Hong Kong, Malaysia, Singapore, Taiwan) to high (China, Japan, Korea, and Vietnam), potentially influencing diagnostic testing and management.^13^, ^14^, ^15^ The clinical characteristics, optimal diagnostic evaluation, and prevalence of organic lesions in patients with dyspepsia in Cambodia have not been previously clarified. Therefore, the objectives of our study were to characterize chronic dyspeptic patients in Cambodia according to symptom groups and to determine the prevalence of *H. pylori*, the risk of organic lesions, and the severity and pattern of gastritis in the different clinical subgroups. We also examined associations between patient characteristics and the presence of *H. pylori* or intestinal parasites and prevalence of organic lesions and determined the performance characteristics of alarm features in predicting organic lesions in this population.

## Methods

This study was approved by the National Ethics Committee for Health Research. We conducted a retrospective, single‐center study of patients seen at an outpatient gastroenterology clinic from May 2011 to March 2015. All patients aged older than 18 years who underwent EGD for evaluation of chronic dyspepsia were identified and analyzed (*n* = 1231). All patients had onset of symptoms at least 6 months before presentation. At each clinic visit, patients were classified as having bothersome epigastric pain or burning, bothersome postprandial fullness or early satiety, or overlapping symptoms of epigastric pain/burning and postprandial fullness/early satiety similar to the Rome IV criteria.^8^ Patients who underwent EGD for indications other than dyspepsia, such as typical gastroesophageal reflux disease (GERD), medical checkup, anemia, cirrhosis, dysphagia, or suspicion of gastrointestinal bleeding, or those who underwent EGD for follow up of known organic lesions (e.g. control of gastric ulcer, evaluation of success of *H. pylori* treatment, follow up of diagnosed malignancy) were excluded. We also excluded patients with less than 3 months’ duration of dyspepsia, who took nonsteroidal anti‐inflammatory drugs (NSAIDs) or aspirin, or who had incomplete data.

Organic lesions were defined as the presence of ulcers (defined as mucosal break of 3 mm or greater) or cancer (confirmed by histology). Erosions (defined as mucosal defect less than 3 mm) were not considered organic lesions in the context of chronic dyspepsia as they could be acute lesions caused by *H. pylori* infection or by NSAID or aspirin intake. We also did not consider peptic esophagitis an organic lesion as gastroesophageal reflux can coexist with functional dyspepsia. Patients with a normal EGD without *H. pylori* on biopsy were diagnosed with functional dyspepsia. Patients were diagnosed with *H. pylori*‐associated dyspepsia if they had a normal EGD with *H. pylori* identified on biopsies or if they had chronic active gastritis without evidence of *H. pylori*.

Patient data were extracted from the medical records in a standardized format including age; gender; symptoms and their classification, such as epigastric pain/burning, postprandial fullness/early satiety, or overlapping symptoms; and presence of alarm signs, endoscopic findings, and specific histological findings. Alarm features were defined as unintentional weight loss of more than 5% of usual body weight over 6–12 months, persistent vomiting, iron deficiency anemia, epigastric mass or lymphadenopathy, dysphagia, odynophagia, jaundice, gastrointestinal bleeding, or family history of upper gastrointestinal cancer.^10^ All patients undergoing EGD had endoscopic biopsies taken, including two samples from the antrum, two samples from the fundus, and one sample from the duodenum. All samples were evaluated for the type and grade of gastritis and the presence of *H. pylori*. The severity of gastritis was graded by the updated Sydney Classification.^16^

### *Statistical analysis*

SPSS version 20.0 was used for the descriptive and comparative analyses. Chi‐square test and *Z*‐test were used to compare categorical and continuous variables, respectively. The performance characteristics of alarm signs for identifying dyspepsia related to organic lesions were calculated. *P*‐values less than 0.05 were regarded as statistically significant.

## Results

**Figure 1:**
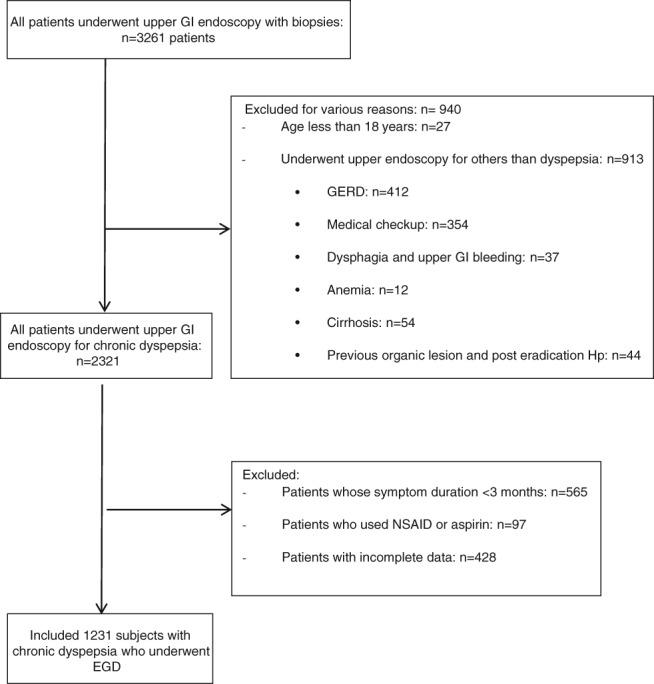
Study flow chart.

A total of 3261 patients underwent outpatient EGD during the study period; 940 patients were excluded because of the presence of symptoms such as typical GERD (*n* = 412), medical checkup (*n* = 354), dysphagia and GI bleeding (*n* = 37), anemia (*n* = 12), cirrhosis (*n* = 54), previous endoscopy of known organic lesion (*n* = 44), and age younger than 18 years of age (*n* = 27) (Fig. 1). We also excluded all patients with a duration of dyspepsia less than 3 months (*n* = 565), who took NSAIDs or aspirin (*n* = 97), and who had incomplete data (*n* = 428). We included 1231 patients with chronic dyspepsia in this study.

### *Demographic characteristics*

Of subjects, 46% were male, with a mean age of 43.5 years (Table 1). Subjects came primarily from Phnom Penh (50.4%). Epigastric pain/burning postprandial fullness/early satiety symptoms were each seen in 29.7%, while 40.6% had overlapping symptoms (Table 1). Belching was seen in 25.6%. Other less common symptoms were nausea/vomiting (9.4%) and upper abdominal bloating (5.8%).

**Table 1:** Baseline subject characteristics

Characteristics	*N* = 1231
Gender (*n*, %)	
Male	570 (46.3%)
Female	661 (53.6%)
Age, years (*n*, %)	
<40	541 (43.9%)
≥40	690 (56.1%)
Geographic origin (*n*, %)	
Phnom Penh	621 (50.4%)
Other provinces	610 (49.6%)
Clinical symptoms (*n*, %)	
Epigastric pain and/or burning	366 (29.7%)
Postprandial fullness and/or early satiety	365 (29.7%)
Overlapping epigastric pain/burning and postprandial fullness/early satiety	500 (40.6%)
Nausea and vomiting	116 (9.4%)
Upper abdominal bloating	72 (5.8%)
Belching	315 (25.6%)

### *Endoscopic and histological findings*

Functional or *H. pylori*‐associated dyspepsia was diagnosed in 93.1%. The major causes of organic dyspepsia were gastric or duodenal ulcers (5.4%) and upper gastrointestinal malignancies (1.5%) (Table 2). Patients with overlapping symptoms were more likely to have a normal EGD (*P* < 0.0001), while patients with only postprandial fullness/early satiety symptoms were more likely to have mucosal erythema or pangastritis (*P* < 0.0001). Gastric or duodenal ulcers were more common in patients with only epigastric pain/burning symptoms (*P* < 0.013) (Table 2).

**Table 2:** Characteristics of different symptom subgroups stratified by *Helicobacter pylori* prevalence and endoscopic and histological outcomes

	Postprandial fullness and/or early satiety (*N* = 365)	Epigastric pain and/or burning (*N* = 366)	Overlapping symptoms (*N* = 500)	*P*‐value
EGD findings (*n*, %)				
Normal	237 (64.9%)	253 (69.1%)	392 (78.4%)	0.0001
Mucosal erythema	81 (22.2%)	48 (13.1%)	54 (10.8%)	0.0001
Gastric/duodenal erosions	25 (6.8%)	26 (7.1%)	23 (4.6%)	0.224
Peptic esophagitis	1 (0.3%)	4 (1.1%)	2 (0.4%)	0.274
Gastric and/or duodenal ulcer	13 (3.6%)	30 (8.2%)	23 (4.6%)	0.013
Upper gastrointestinal tumor	8 (2.2%)	5 (1.4%)	6 (1.2%)	0.479
*Helicobacter pylori* infection	143 (39.2%)	127 (34.7%)	165 (33%)	0.164
Histology (*n*, %)				
Antral gastritis	89 (24.4%)	104 (28.4%)	143 (28.6%)	0.330
Gastritis limited to the fundus	1 (0.03%)	0 (0%)	0 (0%)	NS
Pangastritis	194 (53.2%)	157 (42.9%)	200 (40%)	0.0001

EGD, esophagogastroduodenoscopy.

**Figure 2:**
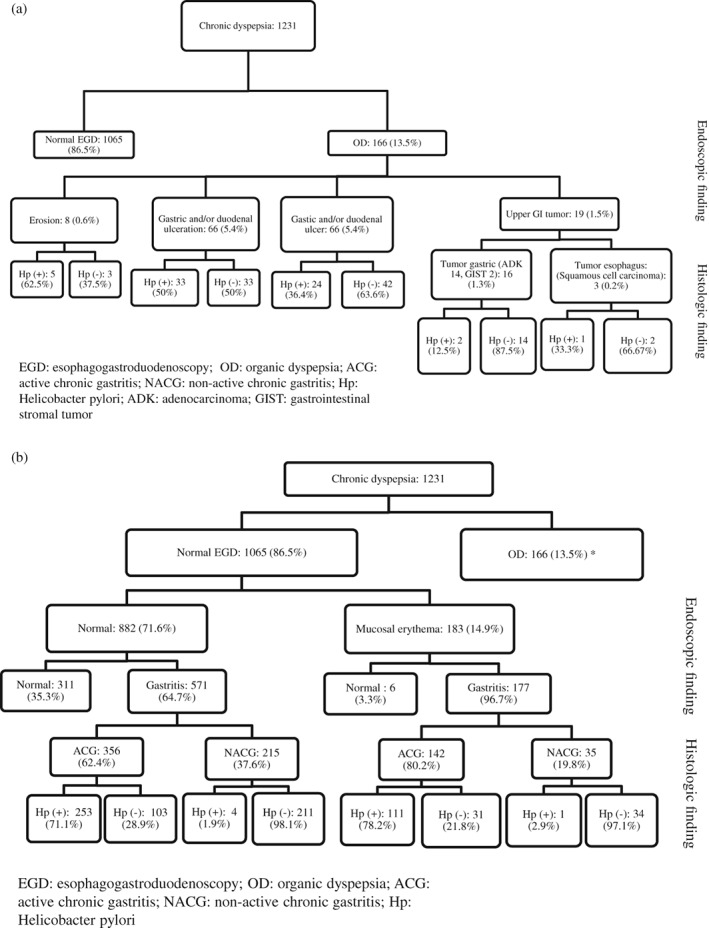
Endoscopic finding, histology results, and prevalence of *Helicobacter pylori* infection; (a) organic dyspepsia; (b) functional dyspepsia and *Helicobacter pylori* associated gastritis.

Of patients, 35% with chronic dyspepsia were diagnosed with *H. pylori*. The prevalence of *H. pylori* was similar in patients with postprandial fullness/early satiety symptoms only, epigastric pain/burning symptoms only, and overlapping symptoms (39.2, 34.7, and 33%, respectively, *P* = 0.16) (Table 2). Of patients with organic dyspepsia, 39% were *H. pylori*‐positive (Fig. 2a). *H. pylori* was found in 29% of dyspeptic patients who had a normal EGD (Fig. 2b) and in 61% of patients with gastric erythema on EGD. Normal gastric mucosa was histologically seen in 30% of patients without organic lesions; all these patients were also negative for *H. pylori*. Of patients without organic lesions endoscopically, 70% had chronic gastritis on histology, 67% of whom had active chronic gastritis (ACG) and 33% nonactive chronic gastritis (NACG); *H. pylori* was found in 73 and 2%, respectively. Pangastritis was seen more commonly in those with postprandial fullness/early satiety symptoms compared to those with epigastric pain/burning or overlapping symptoms (*P* = 0.0001) (Table 2). Atrophic gastritis was found in 29 patients (mean age 51.6 ± 15.9 years), and intestinal metaplasia was found in 45 patients (mean age 49.9 ± 14.0 years). No patient was diagnosed with intestinal parasites on duodenal biopsies.

### *Risk factors for organic dyspepsia*

**Figure 3:**
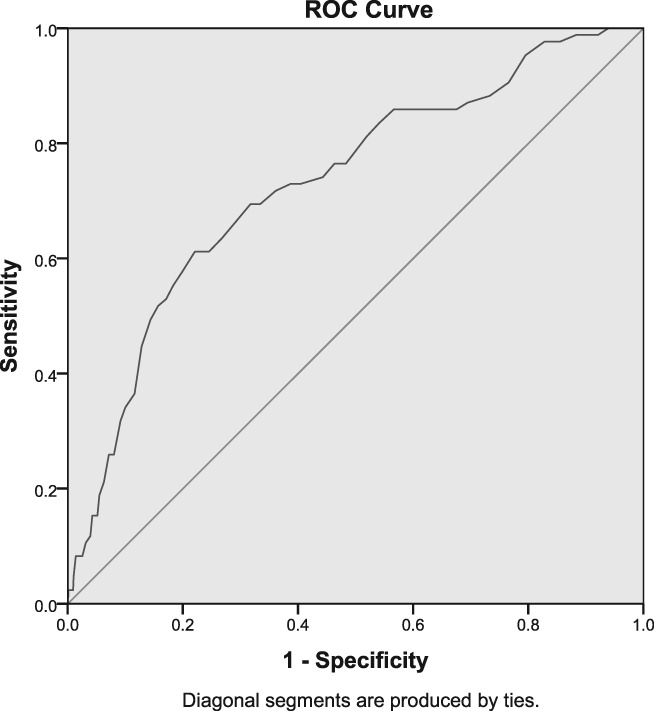
Receiver operator curve for age and diagnosis of organic dyspepsia.

Of patients with organic dyspepsia, 84% were older than 40 years of age, compared to 54% of patients with functional dyspepsia (*P* = 0.001) (Table 3). In univariate analysis, male gender, age older than 40 years, epigastric pain/burning symptoms, and alarm signs were associated with organic lesions. Choosing an age of 40 years as a cutoff resulted in a 85% sensitivity and 55% specificity for the presence of organic dyspepsia (area under the ROC curve = 0.731, *P* < 0.0001) (Fig. 3).

**Table 3:** Risk factors for organic lesions and functional dyspepsia

	Chronic dyspepsia		*P*‐value
	Organic dyspepsia (*N* = 85)	Functional dyspepsia (*N* = 1146)	
Gender (*n*, %)			0.56
Male	48 (56.5%)	522 (45.5%)	
Female	37 (43.5%)	624 (54.5%)	
Age, years (*n*, %)			<0.0001
≥40	71 (83.5%)	620 (54.1%)	
<40	14 (16.5%)	526 (45.9%)	
Clinical symptoms (*n*, %)			
Postprandial fullness and/or early satiety	21 (24.7%)	344 (30.0%)	0.301
Epigastric pain and/or burning	35 (41.2%)	331 (28.9%)	0.017
Overlapping symptoms	29 (34.1%)	471 (41.1%)	0.206
*Helicobacter pylori* present (*n*, %)	27 (31.8%)	408 (35.6%)	0.475
Alarm features present (*n*, %)	12 (14.1%)	46 (4.0%)	0.001

Overall, 4.7% of patients had alarm symptoms, of whom 12 (21%) had organic lesions (Table 3). Among patients without alarm symptoms, 6.2% had organic lesions. The sensitivity of alarm symptoms for organic lesions was 14%, with a specificity of 96%, positive predictive value of 20%, and negative predictive value of 93%.

## Discussion

In our center, functional and *H. pylori*‐associated etiologies of dyspepsia were more common than organic etiologies (93.1% vs. 6.9%). Among those with functional or *H. pylori*‐associated symptoms, the 30% with histologically normal gastric mucosa were all *H. pylori*‐negative; this result was similar to those of the study by Atisook *et al*.^17^ *H. pylori* infection was detected in 73% of those with ACG and 2% with NACG.

We found *H. pylori* infection in 39% of patients with organic dyspepsia, which was similar to its prevalence in those with functional dyspepsia. These results suggest that the presence of *H. pylori* infection should not be used to decide whether a dyspeptic patient should undergo EGD in Cambodia. Furthermore, the utility of noninvasive tests for *H. pylori*, such as the urea breath test and antigen, is questionable as, in our practice, most patients had already taken proton pump inhibitors or antibiotics, potentially affecting test accuracy. In practice, it is difficult for patients to discontinue proton pump inhibitors for 2–4 weeks before noninvasive testing.^14^

Only 3 of 19 patients with gastric cancer had demonstrated *H. pylori* infection. In some of these cases, biopsies from normal mucosa were not performed, potentially affecting diagnostic accuracy. In others, the gastric mucosa may have been atrophic or contained intestinal metaplasia, conditions where *H. pylori* organisms may no longer be viable.^18^ The prevalence of *H. pylori* in our population may also be underestimated because some patients took proton pump inhibitors and/ or antibiotics before EGD. *H. pylori* infection causes ACG in virtually in all infected people, and infection is life‐long unless treated.^19^

We found a slightly higher prevalence of functional dyspepsia compared to many studies,^20^, ^21^, ^22^ which might partially be explained by selection bias as the decision to undergo EGD was based partly by affordability to the patient. Low socioeconomic status is a risk factor for stomach cancer,^14^ and our series likely included patients with relatively high socioeconomic status.

The global prevalence of dyspepsia is overall slightly higher in women compared to men.^23^ In our study, 54% of patients with dyspepsia were women, similar to results seen in an Italian population,^21^ but slightly lower than in a Brazilian population.^22^ This slight female predominance could be explained by the fact that women seek health care earlier than men, even with mild symptoms. Moreover, women are at a higher risk of functional dyspepsia due to biological, gender, and social differences.^24^

The prevalence of organic lesions (ulcer and cancer) was higher in patients older than 40 years of age, and all patients with gastric cancer in this series were older than 40 years of age. The age at risk of gastric cancer in our series was younger than in other countries in Asia. Earlier age of cancer diagnosis in Cambodia might be related to exposure to *H. pylori* infection or to salt and salt‐preserved foods.^14^ In addition, after the Khmer Rouge regime, the majority of Cambodians report their age as 3–5 years younger than their real age to either be accepted for work or to prolong their working period (personal observation). Asian consensus has recommended endoscopy in adults older than 45 years old with persistent dyspepsia [3]. Our findings suggest that endoscopy be considered in Cambodian adults with a reported age of older than 40 years with dyspepsia.

Our results are consistent with previous literature showing that alarm features have poor sensitivity, specificity, and predictive value for distinguishing organic and functional dyspepsia.^1^ For example, Bai *et al*., in China, showed that the sensitivity and specificity of alarm features for upper gastrointestinal malignancy were 13.4 and 96.6%, respectively.^25^ A systematic review by Vakil *et al*. showed that positive and negative predictive values of weight loss for organic lesions varied from 1.5 to 15.8% and 97.6 to 99.7%, respectively, and of dysphagia varied from 1.5 to 7.1% and 97.6 to 99.8%, respectively.^26^ In addition, the positive and negative predictive values of anemia varied from 0 to 4.9% and 96.3 to 99.8%, respectively. This wide variation in performance may be due to the small number of cancer cases detected in many studies and heterogeneity in study settings. Nevertheless, in our setting, the presence of alarm symptoms has limited value in detecting upper gastrointestinal malignancy.

In our series, the proportions of subjects with only epigastric pain/burning or postprandial fullness/early satiety was lower compared with other studies.^2^, ^10^ This might be explained by our retrospective design where available clinical data might not thoroughly differentiate between these three symptom complexes. In addition, patients who reported evolution of symptoms over different clinic visits were classified as having overlapping symptoms. Nevertheless, our study is reflective of clinical practice where patient symptoms vary over time and where classification into a single symptom subgroup may not be possible. Similar to prior studies, there was no significant difference regarding prevalence of *H. pylori* in patients with different symptoms.^21^ We did find significant differences in endoscopic findings between the symptom subgroups. Patients with postprandial fullness/early satiety only had a higher prevalence of gastric erythema, while patients with epigastric pain/burning only were more likely to have gastric or duodenal ulcers. Our results differ from Fang *et al*., who showed that *H. pylori* infection was associated with only PDS (according to Rome IV criteria).^2^ Precancerous lesions such as gastric atrophy and intestinal metaplasia were not associated with any symptom subgroup, again demonstrating poor correlation of symptoms with organic etiologies of dyspepsia.

**Figure 4:**
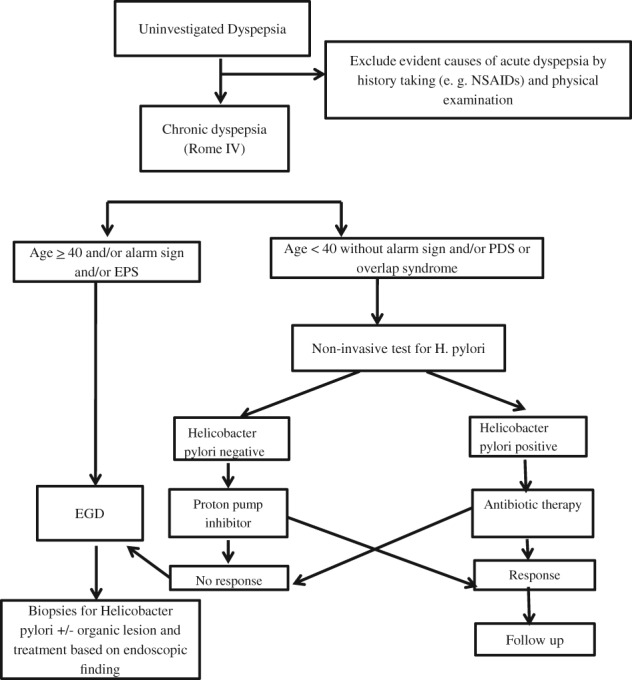
Proposed algorithm for the management of chronic dyspepsia in Cambodia.

In conclusion, the majority of dyspeptic patients in Cambodia were diagnosed with functional or *H. pylori*‐associated dyspepsia. The risk of organic lesions was higher in those aged 40 years and older, and all patients with gastric cancer were older than this cutoff. Overlapping symptoms were predominant and were associated with normal endoscopic findings, whereas postprandial fullness/early satiety and epigastric pain/burning symptoms were associated with mucosal erythema and gastroduodenal ulcers on EGD, respectively. The low sensitivity of alarm features suggests that their absence does not to rule out organic lesions, but their higher specificity indicates that the presence of alarm features can identify patients in whom endoscopic evaluation should be considered. Our results suggest that, in the outpatient setting, if there is no family history of gastric cancer, only patients aged 40 years or older with alarm features or with epigastric pain/burning symptoms should be investigated by EGD to identify organic lesions (Fig. 4). The rapid progress of technologies like image‐enhanced endoscopy might be helpful in detecting precancerous lesions in these groups of patients.^27^, ^28^ Dyspeptic patients younger than the age of 40 years with overlapping symptoms or postprandial fullness/early satiety and without alarm features should be tested for *H. pylori* infection using noninvasive methods and treated if positive, recognizing that patients may have false negative results if they have recently taken antibiotics or proton pump inhibitors.^14^, ^29^ Those who do not have *H. pylori* should be treated for functional dyspepsia with the low risk of missing an organic cause of dyspepsia. This approach will require additional prospective validation in our center and others and may differ according to the prevalence of *H. pylori* and other sociodemographic characteristics of the patient population.
